# Boosting the Electrochemical Performance of PI-5-CA/C-SWCNT Nanohybrid for Sensitive Detection of *E. coli* O157:H7 From the Real Sample

**DOI:** 10.3389/fchem.2022.843859

**Published:** 2022-02-10

**Authors:** Huan Wang, Yanmiao Fan, Qiaoli Yang, Xiaoyu Sun, Hao Liu, Wei Chen, Ayesha Aziz, Shenqi Wang

**Affiliations:** ^1^ Advanced Biomaterials and Tissue Engineering Center, College of Life Science and Technology, Huazhong University of Life Science and Technology, Wuhan, China; ^2^ School of Chemical Science and Engineering Fiber and Polymer Technology, KTH Royal Institute of Technology, Stockholm, Sweden

**Keywords:** poly-5-carboxyindole, carboxylated single-walled carbon nanotubes, *E. coli* O157:H7, electrochemical immunosensor, indole-5-carboxylic acid, plate counting method

## Abstract

Redox activity is an important indicator for evaluating electrochemical biosensors. In this work, we have successfully polymerized indole-5-carboxylic acid into poly-5-carboxyindole nanomaterials (PI-5-CA), using its superior redox activity, and introduced carboxylated single-walled carbon nanotubes (C-SWCNTs) to synthesize a composite material. Finally, a synthesized composite material was used for the modification of the glass carbon electrode to fabricate the PI-5-CA/C-SWCNTs/GCE-based immunosensor and was successfully applied for the sensitive detection of *E. coli* O157:H7. The fabricated immunosensor exhibited an outstanding electrocatalytic activity toward the detection of *E. coli* O157:H7 with a remarkably lowest limit of detection (2.5 CFU/ml, *LOD* = 3 *SD*/k, *n* = 3) and has a wide linear range from 2.98×10^1^ to 2.98×10^7^ CFU/ml. Inspired from the excellent results, the fabricated electrode was applied for the detection of bacteria from real samples (water samples) with a good recovery rate (98.13–107.69%) as well as an excellent stability and specificity. Owing to its simple preparation, excellent performance, and detection time within 30 min, our proposed immunosensor will open a new horizon in different fields for the sensitive detection of bacteria from real samples.

## Highlights

• PI-5-CA/C-SWCNT nanohybrids are synthesized by facile methods
• The PI-5-CA/C-SWCNT nanohybrid-modified GCE was further incubated with the *E. coli* antibody to complete the antigen–antibody reaction to fabricate the Ab/PI-5-CA/C-SWCNTs/GCE immunosensor
• Ab/PI-5-CA/C-SWCNTs/GCE shows an excellent electrochemical activity for *E. coli* O157 detection
• Real-time *in vitro* detection of *E. coli* O157 from real samples and compared results obtained from the actual samples

## 1 Introduction

To date, the most important topic of concern for food industries is the alarming increase of food- and waterborne diseases (Law et al., 2015; Patra and Baek, 2016). According to statistics from the World Health Organization (WHO), up to 30% of the world’s population suffers from foodborne diseases every year (Jia and Jukes, 2013). Factors that cause foodborne diseases include bacteria, parasites, viruses, chemicals, and toxins (Aziz et al., 2021; Rad et al., 2021). Among these factors, bacterial contamination is an alarming threat to human health (Chen et al., 2017; Sai-Anand et al., 2019). Bacteria are ubiquitous in nature, and bacterial contamination may occur in any food chain (Odeyemi et al., 2020). If food chains once get infected with these pathogens, it can seriously threaten human health and can cause economic losses, if not treated timely (Asif et al., 2018). In 2011, there was an outbreak in the United States due to the contamination of cantaloupe instigated by *Listeria monocytogenes*, which infected 147 with 33 deaths (Ghosh et al., 2019). In the same year, Germany also experienced a massive outbreak of hemolytic uremic syndrome, which was initiated by *E. coli* O104:H4 infection (Stockman, 2013). Above all, every year, the number of infections caused by *Salmonella* crossed one million, leading to severe illness and sometimes death (Jarvis et al., 2016). In 2016, 13 cases of diarrhea occurred in nine U.S. states due to the consumption of flour infected with *E. coli* O157:H7 (Sperber and North American Millers' Association Microbiology Working Group, 2007). Therefore, fast and reliable detection of pathogens is essential to prevent and control the outbreaks of foodborne diseases.

Among the common pathogens in daily life, *E. coli* O157:H7 is one of the most hazardous foodborne pathogens because of its virulence and pathogenicity (Buchanan and Doyle, 1997; Zhao et al., 2021). Diseases caused by *E. coli* O157:H7 include diarrhea, fever, and vomiting (Pandey et al., 2017). At present, quite a lot of attention has been devoted to the research for the rapid detection of *E. coli* O157:H7 (Park et al., 2020) The conventionally used plate counting method is reliable to some extent but inevitably limited owing to the time-consumption (Sieuwerts et al., 2008; Zhao et al., 2014). Technological advances introduced and proposed new methods and techniques, such as polymerase chain reaction (PCR) (Amagliani et al., 2004; Zhou et al., 2022) and enzyme-linked immunosorbent assay (ELISA) (Di Febo et al., 2019; Hu Y. et al., 2021), but the requirement of high precision and accuracy as well as the need of highly professional trainers limited their use to some extent. To address all these issues related to conventional and advanced techniques, biosensors have been developed (Aziz et al., 2022). The development of biosensors can solve the abovementioned problems (Asif et al., 2019; Aziz et al., 2019b), such as colorimetry (Yao et al., 2020), fluorescence (Shi et al., 2015), and electrochemistry (Li et al., 2021). Among them, the electrochemical method has received widespread attention because of the low cost, easy handling, and portability (Asif et al., 2022).

Many electrochemical redox active materials have been used as electronic media for the development of electrochemical biosensors, such as ferrocene (Hu L. et al., 2021), graphene oxide (GO) (Aziz et al., 2019a), and Prussian blue (22). However, most of these materials suffer low conductivity and poor stability, so their effects in the field of electrochemical detection are not satisfactory (Kang et al., 2016). As a conductive polymer, poly (indole-5-carboxylic acid) (PI-5-CA) exhibits good electrochemical behavior, good thermal stability, and superior redox activity due to its abundant functional groups and specific surface area (Asif et al., 2015; Yang et al., 2019). At the same time, the introduction of carboxylated single-walled carbon nanotubes (C-SWCNTs) can further improve the specific surface area and the electrical conductivity of PI-5-CA. Due to its tubular hollow structure, carbon nanotubes have unique electrical conductivity, high strength, flexibility, stable chemical properties, and excellent specific surface area (Kumar and Sundramoorthy, 2019; Li et al., 2021). Through chemical synthesis, PI-5-CA and C-SWCNTs are synthesized into a composite material to syndicate the electrochemical advantages of the two, and using their abundant carboxyl functional groups to combine with various biological recognition molecules (Yang et al., 2019).

**FIGURE 1:**
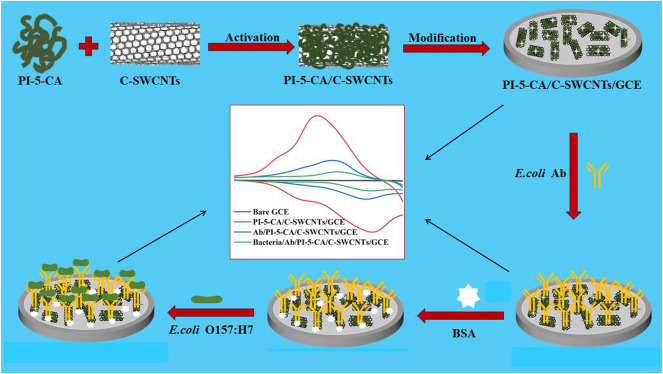
Schematic illustration of the step-by-step preparation of PI-5-CA/C-SWCNTs/GCE and its modification with antibodies and BSA for the sensitive detection of *E. coli* O157:H7.

Therefore, we use the superior electrical conductivity of C-SWCNTs and the ultrahigh redox activity of PI-5-CA to construct an electrochemical sensing platform (Joshi and Prakash, 2013; Yang et al., 2019). At the same time, we use the characteristic of antigen-antibody-specific binding to propose an electrochemical immunosensor to detect *E. coli* O157:H7. First, the PI-5-CA/C-SWCNT composite material was synthesized for the modification of glassy carbon electrode (GCE), and the redox characteristics of the material were explored using the classic three-electrode system. By activating the carboxyl group on the surface of the material and binding with the amino group of the antibody, the anti-*E. coli* antibody is connected to the surface of the modified GCE for *E. coli* O157:H7 detection as represented by Figure 1. In this research work, PI-5-CA was used to provide a stable redox signal to improve the detection sensitivity (Joshi and Prakash, 2013), while C-SWCNT coupling was used to further improve stability and conductivity (Joshi and Prakash, 2013), as well as provide abundant binding sites for antibodies, which in turn ensure the detection specificity. By detecting the change of PI-5-CA redox current, the rapid and sensitive detection effect of *E. coli* O157:H7 is realized (Yang et al., 2021). We used this constructed biosensor to successfully detect *E. coli* in domestic water, and compared the results with the traditional culture method to determine the sensitivity and reliability of the fabricated sensor.

## 2 Experimental Sections

### 2.1 Chemicals and Reagents

Indole-5-carboxylic acid (I-5-CA) was purchased from Shanghai Vita Chemical Regent Co., Ltd. (Shanghai, China). Carboxylated single-walled carbon nanotubes (C-SWCNTs) were purchased from Nanjing Xian Feng Nanomaterials Technology Co., Ltd. (Nanjing, China). N-hydroxysuccinimide (NHS) and N-(3-dimethylaminopropyl)-N′-ethylcarbodiimide hydrochloride (EDC) were purchased from Aladdin Chemistry Co., Ltd. (Shanghai, China). Bovine serum albumin (BSA) and 2-morpholinoethanesulfonic acid (MES) were purchased from Sigma-Aldrich (United States). The anti-*E. coli* O157:H7 antibody was purchased from Thermo (United States). Ethanol, ammonium persulfate (APS), disodium hydrogen phosphate (Na_2_HPO_4_), sodium dihydrogen phosphate (NaH_2_PO_4_), and H_2_SO_4_ were purchased from Sinopharm Chemical Co., Ltd. (Shanghai, China). All the chemicals and reagents were used as it is, without further purification. The four different strains were used in this research as shown in Table 1 **.**

**TABLE 1:** Information of the strains used in this work.

Bacteria	Strain number
*E. coli* O157:H7	CCTCC AB 200051
*Staphylococcus aureus*	CCTCC AB 2013186
*Salmonella typhimurium*	CCTCC AB 204062
*Pseudomonas aeruginosa*	ATCC27853

### 2.2 Synthesis of PI-5-CA/C-SWCNTs

The PI-5-CA/C-SWCNT nanocomposite was synthesized by the chemical method. First, 100 mg of In-5-COOH monomer and 2 mg of carboxylated single-walled carbon nanotubes (C-SWCNTs) were dissolved in 2.5 ml of absolute ethanol, Next, 100 mg ammonium persulfate (APS) was dissolved in 10.0 ml of H_2_SO_4_ (pH = 1). Under constant temperature stirring, the mixed solution of 100 mg ammonium persulfate (APS) dissolved in 10 ml H_2_SO_4_ (pH = 1) was added gradually, and the mixture was left to react at 30°C for 6 h. After the reaction was completed, the product was filtered and washed with ultrapure water and absolute ethanol several times in sequence. Finally, we used this solid product to prepare 1 mg/ml solution in ultrapure water for further use.

### 2.3 Fabrication of the Electrochemical Immunosensor

Before each experiment, the glassy carbon electrode (3 mm in diameter) was polished to a mirror surface with 0.05 μM alumina powder and ultrasonically treated with ultrapure water and absolute ethanol, respectively. Finally, the cleaned electrode was dried with high-purity nitrogen for the next modification.

For the modification of electrode, 10 µL of 1 mg/ml solution of PI-5-CA/C-SWCNTs was injected onto the surface of the GCE and dried in air naturally. To activate the carboxyl group on the composite material, first, the modified electrode was immersed in a mixed solution (containing 40 mM NHS, 100 mM EDC and 100 mM MES), and then incubated at 37°C for 30 min accompanied by the subsequent deposition of 10 μL of Ab solution (5 μg/ml). After that the prepared Ab/PI-5-CA/C-SWCNTs/GCE was further incubated at 37°C for 2 h to ensure that the antibodies bind to the electrode surface. Next, 10 µL of BSA (1 mg/ml) was added dropwise onto the electrode surface and incubated at 37°C for 30 min to block the residual active sites. Finally, the prepared immunosensor was successfully used against bacterial detection and repeated the same procedure after each experiment. It is noted that after each modification, the electrode should be gently washed with PBS (pH = 6) to remove physical adsorption (26).

### 2.4 Preparation of Samples

In order to obtain satisfactory results, pretreatment of the bacterial culture medium is necessary. The bacterial strains used in the research were inoculated into the 5 ml LB medium and cultured at 37°C and 200 rpm for 6 h to their logarithmic growth phase. After that, the freshly cultivated bacterial liquids were centrifuged and immersed in PBS to further dilute into appropriate concentrations. The sample preparation process for actual testing is as follows: first, the tap water was filtered three times with a 0.22 µM filter membrane, and then freshly cultured *E. coli* O157:H7 was added to obtain a natural sample.

### 2.5 Analytical Performance of the Immunosensor

First, 10 µL of the above bacterial liquid was injected to the surface of the immunosensor electrode, and it was incubated at 37°C for 2 h to complete the antigen–antibody reaction. After that, the electrode was gently washed with PBS (pH = 6) to remove the physical absorption. All electrochemical experiments were performed on a CHI660A electrochemical workstation. Throughout the electrochemical experimentation, a three-electrode system (Ag/AgCl as the reference electrode, platinum plate as the counter electrode, and modified electrode as the working electrode) was used to perform cyclic voltammetry (CV) under −0.2–0.8 V at a scanning speed of 100 mV/s to evaluate the electrode surface behavior. Throughout the experimentations, PBS (pH = 6) was used.

## 3 Characterization of the PI-5-CA/C-SWCNTS Composite

**FIGURE 2:**
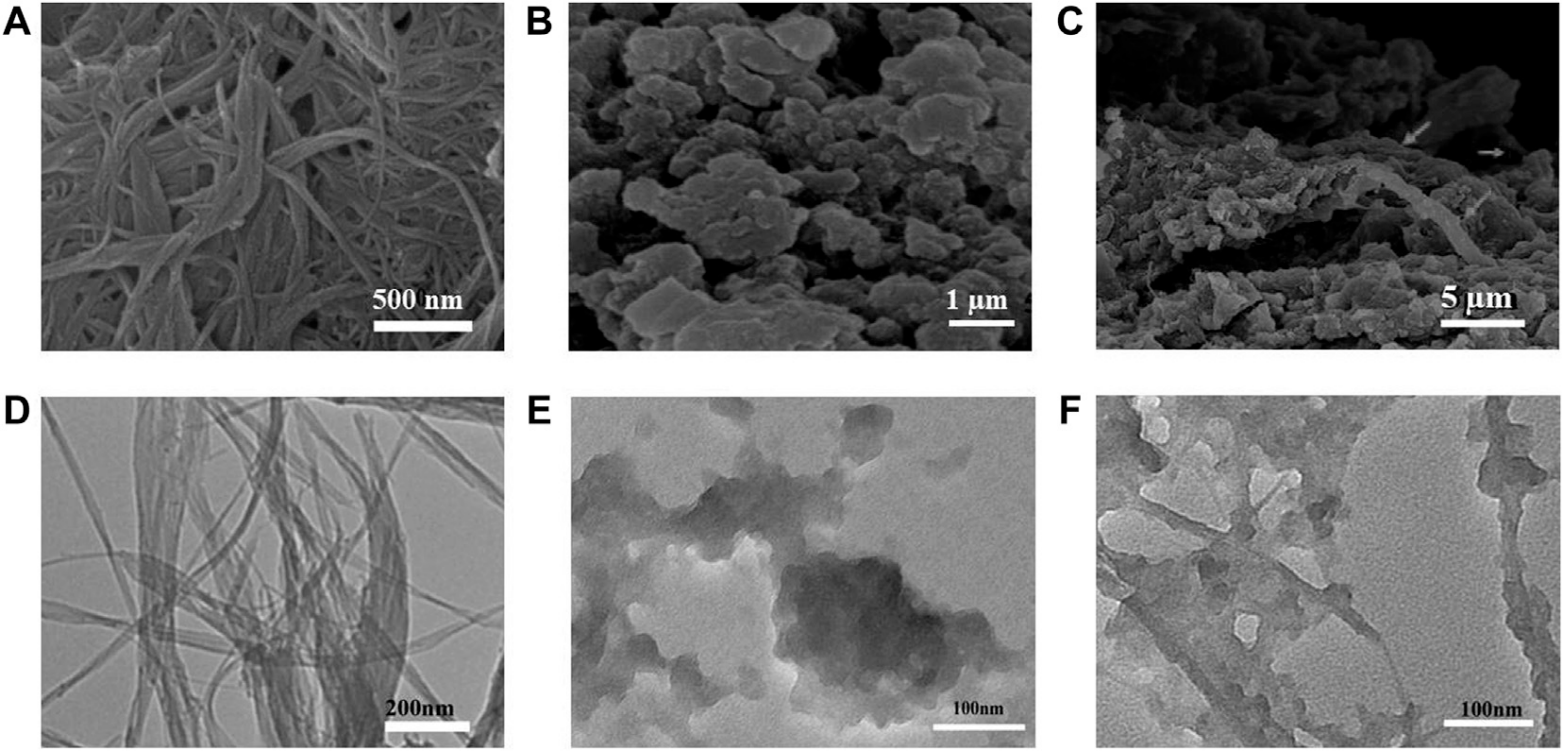
**(A–C)** SEM images of C-SWCNTs, PI-5-CA, and PI-5-CA/C-SWCNTs at different magnifications, and TEM images of **(D)** C-SWCNTs, **(E)** PI-5-CA, and **(F)** PI-5-CA/C-SWCNTs.

The surface morphologies of PI-5-CA and C-SWCNTs were initially characterized by using the transmission electron microscope (TEM) and scanning electron microscope (SEM) to observe the morphology of the three-dimensional structure of PI-5-CA/C-SWCNTs. The SEM images of PI-5-CA/C-SWCNT nanocomposite at different magnifications showed in Figures 2A–C formed a three-dimensional layered porous structure, which can facilitate the combination of various biorecognition molecules and improve the analytical performance of the electrochemical sensor based on PI-5-CA/C-SWCNTs. PI-5-CA exhibited a distinct aggregated morphology that was further combined with SWCNTs to form a distinct three-dimensional structure. SWCNT rods covered with aggregated PI-5-CA as rough surfaces greatly enhance the surface area to provide more active sites to complete the catalytic reaction. SWCNTs support completely burying inside PI-5-CA enhances the catalytic efficiency of the fabricated material being conductive materials. The SEM image results are also consistent with the TEM results taken at different magnification as shown in Figures 2D–F.

**FIGURE 3:**
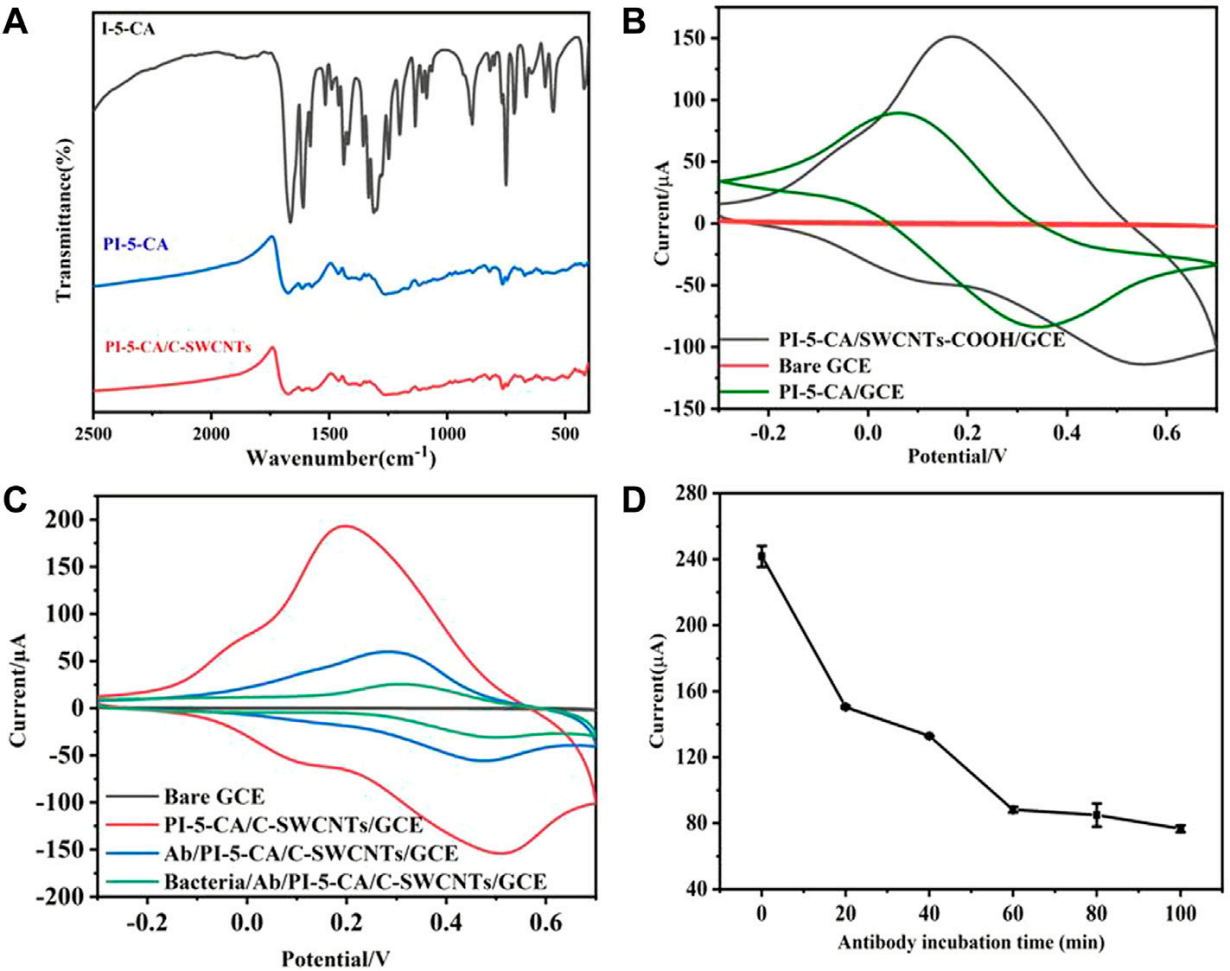
**(A)** FT-IR characterization results of I-5-CA, PI-5-CA, and PI-5-CA/C-SWCNTs. **(B)** CV of electrodes modified with PI-5-CA/C-SWCNTs, bare GC, and PI-5-CA in 0.1 M PBS (pH = 6). **(C)** CV representation of the electrode in 0.1 M PBS (pH = 6) after each step of modification, and **(D)** the current changes of the immunosensor under different antibody incubation times in 0.1 M PBS (pH = 6).

The polymerization mechanism of PI-5-CA was studied by Fourier transform infrared spectroscopy (FT-IR), and the result is shown in Figure 3A. The spectral absorption peak intensity of PI-5-CA is significantly wider than that of I-5-CA monomer, which may be attributable to the wide conjugated chain length distribution of polymers (Liu et al., 2016; Yang et al., 2021). Among them, in the spectrum of monomer I-5-CA and polymer PI-5-CA, the fluctuation of absorption peak in the range of 700–820 cm^−1^ is caused by the deformation vibration of three C-H on the benzene ring, which indicates that the polymerization of monomer occurs on the pyrrole ring (Sivakkumar et al., 2005). The = CH-N stretching vibration of the monomer near 890 cm^−1^ disappeared in the polymer spectrum. The peaks near 1,478–1,838 cm^−1^ showed the presence of carboxyl groups in the monomer I-5-CA and the polymer PI-5-CA (Narang et al., 2013). Compared with the FT-IR spectra of PI-5-CA, the FT-IR spectra of PI-5-CA/C-SWCNT nanocomposites showed a one-point positive shift in the C=C bond, which should be attributed to the π–π interaction between PI-5-CA and C-SWCNTs (Yang et al., 2019).

## 4 Results and Discussion

### 4.1 Electrochemical Performance of PI-5-CA/C-SWCNTs/GCE

Compared with the bare GCE, both PI-5-CA/C-SWCNTs and PI-5-CA-modified electrodes can promote electron transfer and generate redox current. It can be seen from Figure 3B that the redox current peak value of PI-5-CA/C-SWCNTs/GCE is significantly higher than that of PI-5-CA/GCE, which may be attributed to the tubular structure of C-SWCNTs promoting the electron transfer of PI-5-CA. Figure 3C shows the electron transfer behavior of the electrode surface after each modification. Because PI-5-CA/C-SWCNTs have higher redox activity and conductivity, a higher redox current peak can be clearly seen. In that case, when antibodies bound to the carboxyl groups present on the surface of PI-5-CA/C-SWCNTs, a reduction in the peak redox current can be clearly observed. This is due to the fact that the antibody protein is a non-conductive substance, which causes hindrance during the electron transfer on the electrode surface that resultantly causes a decrease in redox current. After incubating with *E. coli* O157:H7 bacterial solution, the peak redox current further decreased, which just proved that the bacterial solution successfully combined with the antibody on the electrode surface. This reduction was due to the increased steric effect of antigen–antibody immune complexes during electron transfer. The current change on the surface of the glassy carbon electrode indicates the successful preparation of the immunosensor, which can be further used as a potential platform for detecting bacteria.

### 4.2 Optimization of Experimental Parameters

#### 4.2.1 Optimization of Antibody Incubation Time

When the PI-5-CA/C-SWCNT composite material was deposited on the surface of the electrode, the carboxyl group can be activated with a mixed solution containing EDC, NHS, and MES. Then, 10 µL of antibody solution was dropped onto the surface of the electrode, which helps antibodies to get attached to the surface of the GCE through the amino-carboxyl reaction in a 37°C water bath. In order to make sure the -COOH group of the PI-5-CA/C-SWCNT composite material can bind to more and more antibodies, the incubation time was optimized within 100 min.

It can be seen from Figure 3D that as the incubation time increases, the peak value of the redox current on the electrode surface gradually decreases. This is due to the gradual increase in the amount of antibodies bound to the electrode surface, which increases the impedance of electron transfer. As shown in Figure 3D, after about 60 min of reaction, the current peak gradually stabilized. It can be concluded that the antibodies bound to the electrode surface reach a relatively saturated state after 60 min of incubation. Later studies also chose 60 min as the antibody incubation time.

#### 4.2.2 Optimization of Incubation Time for *E. coli* O157:H7

In order to ensure the binding of sufficient amount of bacteria on the electrode to achieve a sensitive detection effect, the incubation time of the bacteria solution was further optimized. The freshly cultured *E. coli* O157:H7 bacterial solution was immersed in PBS after centrifugation, and then was diluted to different concentrations. In total, 10 µL of 4 × 10^6^ CFU/mL *E. coli* O157:H7 bacterial liquid was added dropwise to the prepared immunosensing electrode and incubated at 37°C for different times.

**FIGURE 4:**
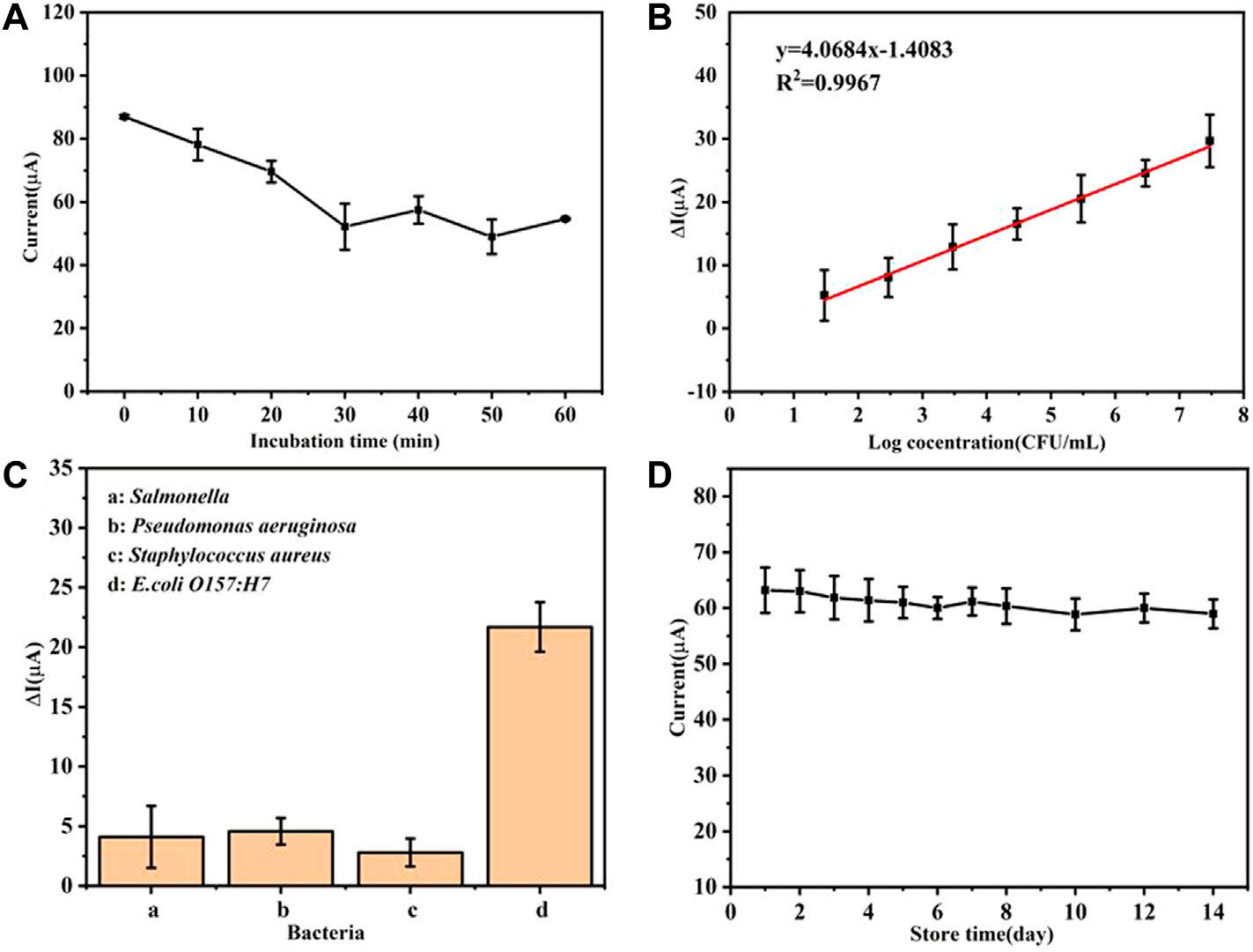
**(A)** Current changes of the immunosensor under different bacterial incubation times, **(B)** linear relationship between the current change value and the logarithm of the bacterial concentration, **(C)** detection specificity of the immunosensor, and **(D)** performance of the electrochemical immunosensors at various storage periods.

As the incubation time increases, the oxidation peak current gradually decreases and the current value tends to stabilize at about 30 min as depicted by Figure 4A. In the subsequent incubation time, the fluctuation of the current value may be attributed to the reversibility of the antigen–antibody immune binding reaction (Ghosh, 2006). In summary, 30 min was selected as the reaction time for the combination of bacteria and immunosensor.

### 4.3 Analytical Performance of the Immunosensor

Under the optimal experimental conditions, the analytical performance of the prepared immunosensor was studied. The *E. coli* O157:H7 monoclonal colony was picked into the LB liquid medium and cultivated to a logarithmic phase at 37°C with 200 rpm shaking. Later, the freshly cultured bacterial solution was centrifuged and immersed in PBS. Finally, the bacterial liquid was diluted to a series of concentration gradient from 2.98×10^1^ to 2.98 × 10^7^ CFU/ml, and 10 µL of the abovementioned diluted bacterial solution was dropped onto the electrode surface and incubated for 30 min in a 37°C water bath. After that, the current changes on the electrode surface were recorded.

It can be seen from Figure 4B that there is a good linear relationship between the current change value (ΔI) (before and after the immunosensor is combined with the bacterial solution) and the logarithmic value of the bacterial solution concentration [Log(CFU/ml)]. After fitting, within the linear range, the linear relationship between ΔI and the concentration of *E. coli* O157:H7 is ΔI = 4.0684 Log(CFU/mL)-1.4083 (*R* ^2^ = 0.9976) with a low limit of detection of 2.5 CFU/ml (*LOD* = 3 *SD*/k, *n* = 3). It can be concluded that the prepared immunosensor platform has great potential for the rapid detection of *E. coli* O157:H7, which can provide a basis for the next step of detection in natural samples.

### 4.4 Analytical Specificity of the Immunosensor

In order to explore specificity of the biosensing system for *E. coli* O157:H7 detection, different types of strains such as *Salmonella*, *Pseudomonas aeruginosa*, *Staphylococcus aureus*, and *E. coli* O157:H7 have been detected by repeating the same detection procedure. In total, 10 µL of 10^6^ CFU/ml of the aforementioned various fresh bacterial liquids were injected onto the prepared immune-electrode surface, respectively, for the sensitive strain detection.

In comparison to the *E. coli* O157:H7 modified immunosensor, the other immunosensors modified with *Salmonella*, *Pseudomonas aeruginosa*, and *Staphylococcus aureus* exhibited up to 20% less response toward the target strain as shown in Figure 4C. It can be seen that the specificity of the developed electrochemical immunosensor is acceptable.

### 4.5 Stability of the Immunosensor

The storage performance of this sensor also was studied. The prepared antibody sensor was stored at 4°C and the peak value of the redox current on the electrodes was detected every other day. It can be seen from Figure 4D that the prepared immunosensor has good storage stability. After 1 week, it can still maintain 96.78% of the original current value. After 2 weeks of storage, the current value was about 93.30% of the original value, which further shows that the sensor has good stability and practical applications potential.

### 4.6 Detection and Analysis of Real Samples

Benefitted from the excellent electrochemical performance, we applied our modified electrode for the detection of *E. coli* O157:H7 from the real samples and compared the results obtained from the actual samples with the plate counting method in Table 2.

**TABLE 2:** Assay results of the actual sample using the proposed and plate counting method.

Added (CFU/mL)	Detected (CFU/mL)		Recovery (%)	
	Biosensor	Plate count	Biosensor	Plate count
1.04×10^2^	1.12×10^2^	1.08×10^2^	107.69	103.85
3.2 × 102	3.21 × 102	3.43 × 102	100.31	107.19
3.2×10^4^	3.14×10^4^	3.16×10^4^	98.13	98.75
3.2×10^6^	3.32×10^6^	3.19×10^6^	103.75	99.69

Recovery (%) is expressed as the ratio of the number of detected/number of spiked. As shown in Table 2, the recovery rate of the prepared biosensor is 98.13–107.69%, indicating that the proposed immunosensor for *E. coli* O157:H7 detection has good accuracy. In other words, this electrochemical immunosensor provides a potential application prospect for the analysis of *E. coli* O157:H7 in natural samples.

## 5 Conclusion

In summary, we have successfully proposed a PI-5-CA/C-SWCNT-based electrochemical immunosensor for the rapid detection of *E. coli* O157:H7. First, we prepared PI-5-CA/C-SWCNT composites with a three-dimensional porous structure through a simple chemical oxidation polymerization method. The PI-5-CA/C-SWCNT material has a stable redox activity, good conductivity, large specific surface area, and abundant functional groups. By taking the advantage of these superb characteristics, we used antibodies as biorecognition molecules to construct Ab/PI-5-CA/C-SWCNTs/GCE immunosensing electrodes for the sensitive detection of *E. coli* O157:H7. Compared with previous reported works (Xue et al., 2020; Yang et al., 2020; Qaanei et al., 2021), our fabricated biosensor can detect bacteria as low as 29.8 CFU/ml within 30 min, which greatly shorten the detection time. At the same time, the immunosensor shows good sensitivity, specificity, reproducibility, and stability toward the detection of *E. coli* O157:H7. We believe that the bacteria detection method proposed in this article has good application prospects, which can not only be used for the sensitive and selective detection of *E. coli* O157:H7 but also pave a way for the simple and fast detection of different bacterial strains as well as other substances.

## Data Availability

The original contributions presented in the study are included in the article/Supplementary Material; further inquiries can be directed to the corresponding authors.
